# Investigation of water bonding status of normal and psoriatic skin in vivo using diffuse reflectance spectroscopy

**DOI:** 10.1038/s41598-021-88530-y

**Published:** 2021-04-26

**Authors:** Chao-Chun Yang, Yun-Yo Yen, Chao-Kai Hsu, Nan-Yu Cheng, Shih-Yu Tzeng, Shih-Jay Chou, Jun-Ming Chang, Sheng-Hao Tseng

**Affiliations:** 1 Department of Dermatology, National Cheng Kung University Hospital, College of Medicine, National Cheng Kung University, Tainan, 701 Taiwan, R.O.C.; 2 Department of Photonics, National Cheng Kung University, Tainan, 701 Taiwan, R.O.C.; 3 Instrument Technology Research Center, National Applied Research Laboratories, Hsinchu, 300, Taiwan, R.O.C.

**Keywords:** Applied optics, Skin diseases

## Abstract

Psoriasis affects more than 125 million people worldwide, and the diagnosis and treatment efficacy evaluation of the disease mainly rely on clinical assessments that could be subjective. Our previous study showed that the skin erythema level could be quantified using diffuse reflectance spectroscopy (DRS), and the hemoglobin concentration of most psoriatic lesion was higher than that of its adjacent uninvolved skin. While the compromised epidermal barrier function has been taken as the major cause of clinical manifestation of skin dryness and inflammation of psoriasis, very few methods can be used to effectively evaluate this function. In this study, we investigate the near infrared spectroscopic features of psoriatic (n = 21) and normal (n = 21) skin that could link to the epidermal barrier function. From the DRS measurements, it was found that the water bonding status and light scattering properties of psoriasis are significantly different from those of uninvolved or normal skin. The connection between these parameters to the epidermal barrier function and morphology will be discussed. Our results suggest that objective evaluation of epidermal barrier function of psoriasis could be achieved using a simple DRS system.

## Introduction

Psoriasis is a chronic, inflammatory disease with predominantly skin involvement. It has been found that psoriasis is a systemic inflammatory disease and is often associated with several significant comorbidities including arthritis, cardiovascular diseases, and diabetes^1^. According to the World Health Organization report on psoriasis, psoriasis is a serious global problem with a prevalence rate ranging from 0.09% in Tanzania to 11.4% in Norway^2^.

Although the diagnostic gold standard for inflammatory skin diseases is histopathology, the diagnosis of psoriasis is primarily clinical, and thus relies strongly on the clinicians’ personal judgement. The gross morphological identification and classification of the erythema, thickness and scaling of the psoriatic skin was also important for clinicians to determine the disease severity and initiate a proper treatment procedure.

Typical manifestations of psoriasis include hyperplasia of epidermis, dilated microvasculature of dermis, and compromised skin barrier function. Numerous noninvasive methods have been developed for objective quantification of these phenotypes to assist disease diagnosis. For example, Phillips et al. utilized optical coherence tomography to quantitatively assess epidermal hyperplasia and edema on a psoriatic mouse model^3^. By using laser doppler imaging, Hendriks et al. found alteration in microcirculation of psoriatic lesions was correlated with the skin inflammation status^4^. It has been identified through various studies that the defective barrier function of psoriatic skin can be noninvasively monitored with transepidermal water loss (TEWL) measurements^5,6^. Besides, researchers carried out studies to measure the TEWL and skin impedance of psoriasis, and it was found that the epidermal hydration and barrier function were both decreased compared with normal skin^7,8^.

Diffuse reflectance spectroscopy (DRS) is a model driven optical technique that has been widely applied to investigate the physiological status of various tissue types for its unique features such as simple configuration, cost-effectiveness, and rich spectral information^9–14^. Previously, we reported the use of spatially resolved DRS in quantifying microvasculature and morphology of psoriasis. Specifically, we observed increased hemoglobin concentration in the dermis and increased mean size of tissue scatterers induced by epidermal hyperplasia on psoriasis patients^15^. In psoriatic skin, the content, such as lipids, and proteins, and structure of stratum corneum and living epidermis are disturbed, and thus the water status as well as skin barrier function are affected^16–18^. By using infrared and Raman microspectroscopies, Leroy et al. observed that the definition of skin layer boundaries and the lipid chain order in the stratum corneum were reduced in psoriatic skin as compared to normal skin^16^. In addition, by using Plastic Occlusion Stress Test on psoriatic and normal skin, Fluhr et al. reported that bound water fractions of this two types of skin are distinct^17^. Takenouchi et al. employed the Karl Fischer's method and found that the amount of free water was much smaller in the psoriatic stratum corneum than in the controls^18^. To date, only few reports can be found that employ optical based methods to study the water status of in vivo psoriatic and healthy skin^19,20^. Our goal of this study is to investigate the capability of DRS in deriving the information regarding the status of water molecules in the skin and understand its association to psoriasis.

Water molecules have a strong electrical polarity, and the molecular disposition would be affected by the presence of macromolecules around them. Water molecules that bond the macromolecules such lipids or proteins are called bound water. Researchers have found that the bound water effect introduces modification of water optical absorption spectrum shape^21–23^. This phenomenon was used to noninvasively distinguish malignant breast cancer tissues from normal ones. Due to breast tissue inflammation, it was found that the bound water fraction was lower in malignant breast tissues than in normal ones^24^. Since the cellular and molecular conditions of psoriatic skin are distinct from those of normal skin, we are interested in investigating the water optical absorption spectrum modification introduced by the disturbed molecular condition in psoriatic skin. In this study, we propose a simple and effective method to analyze the near infrared absorption spectra as well as the bound water status of in vivo skin. We first validated the capability of our method in identifying the bound water using a tissue phantom study. The clinical in-vivo measurement revealed that, for the first time, the light absorption spectra of psoriasis and normal skin near water absorption overtones were distinct. Besides, several parameters derived from our DRS system could effectively reflect the skin condition changes in psoriasis, and the diagnostic implication of these parameters will also be discussed.

## Results

### Phantom measurement results

**Figure 1:**
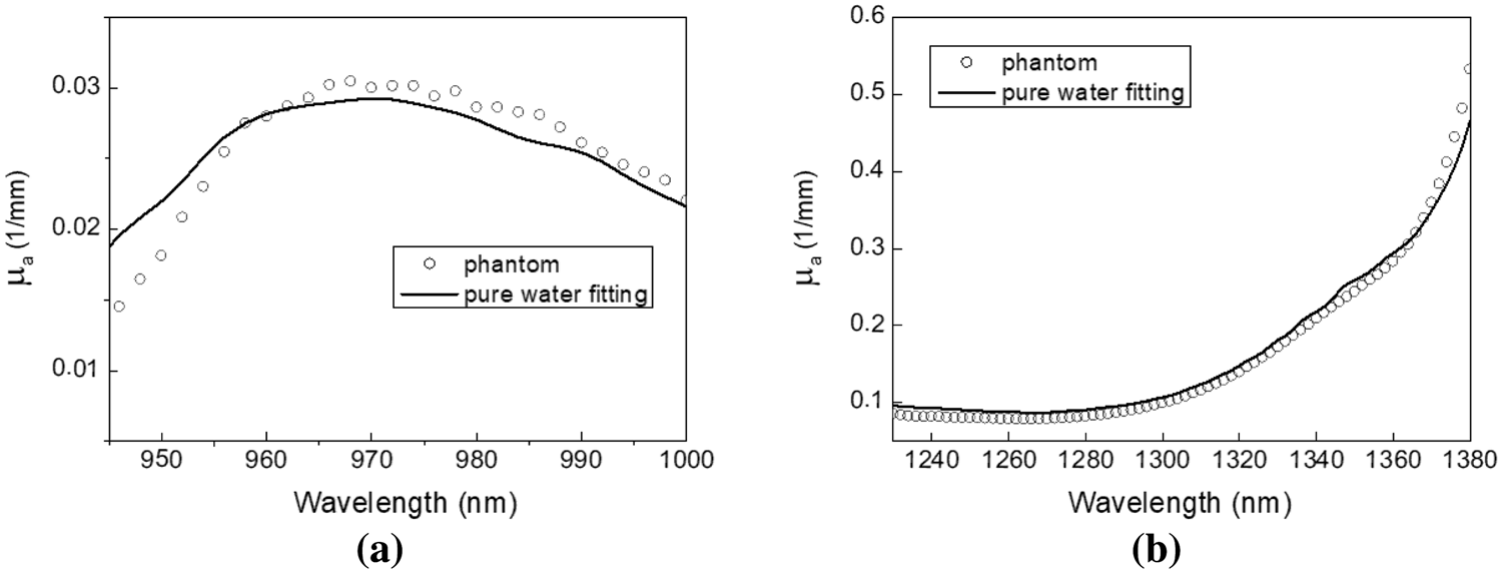
Absorption spectra of the bound water phantom at the first and second water absorption overtone bands. Measured absorption spectra (dotted lines) and the best pure water absorption spectrum fit to them (solid lines) at (**a**) 945–1000 nm, and (**b**) 1230–1380 nm are displayed.

The absorption spectra of the bound water phantom near the second overtone and first overtone of pure water absorption peaks at 25 ℃ were determined using the inverse adding doubling method and are shown in Fig. 1a,b, respectively, as dotted lines. Due to high water absorption strength and the sensitivity limitation of our spectrometer, data collected at wavelengths longer than 1380 nm were noisy and could not be used for calculating the absorption coefficient. We used the pure water absorption spectrum to fit the bound water phantom absorption spectra, and the results are shown as solid lines in Fig. 1. It can be seen in Fig. 1 that in the wavelength ranges between 945 and 1000 nm as well as between 1230 and 1360 nm, the bound water phantom absorption spectrum has a discernable difference from the pure water absorption spectrum. Herrera-Gómez et al. showed that the FWHM (full width at half maximum) of water absorption band at 6060 nm increased with slightly decreased peak wavelength as the amount of bound water increased^23^. Likewise, Eneh et al. showed that the water absorption spectrum at the wavelength of 3900 nm was blue shifted and broadened as bound water fraction increased^22^. Chung et al. reported that at 975 nm the bound water absorption spectrum was red shifted as compared to the pure water absorption spectrum^24^. Our measurement result at the second water absorption overtone region, depicted in Fig. 1a, has a red shifting effect similar to that described in the Fig. 1c of ref^24^. On the other hand, it can be seen in Fig. 1b that the bound water phantom absorption spectrum between 1360 and 1380 nm is blue shifted from the pure water absorption spectrum which is similar to the phenomenon noted by Eneh et al. at around 3900 nm^22^. This could be caused by the first overtone absorption peak broadening. It is worth noting that we fabricated another Lipofundin phantom which had no gelatin powder added, and its absorption spectrum was almost identical to the pure water absorption spectrum (data not shown). Our phantom study results elucidate that the bound water effect induces absorption spectrum deviation from the pure water absorption spectrum both at the first and second overtones.

### Human skin measurement results

#### Typical psoriasis absorption spectra

**Figure 2:**
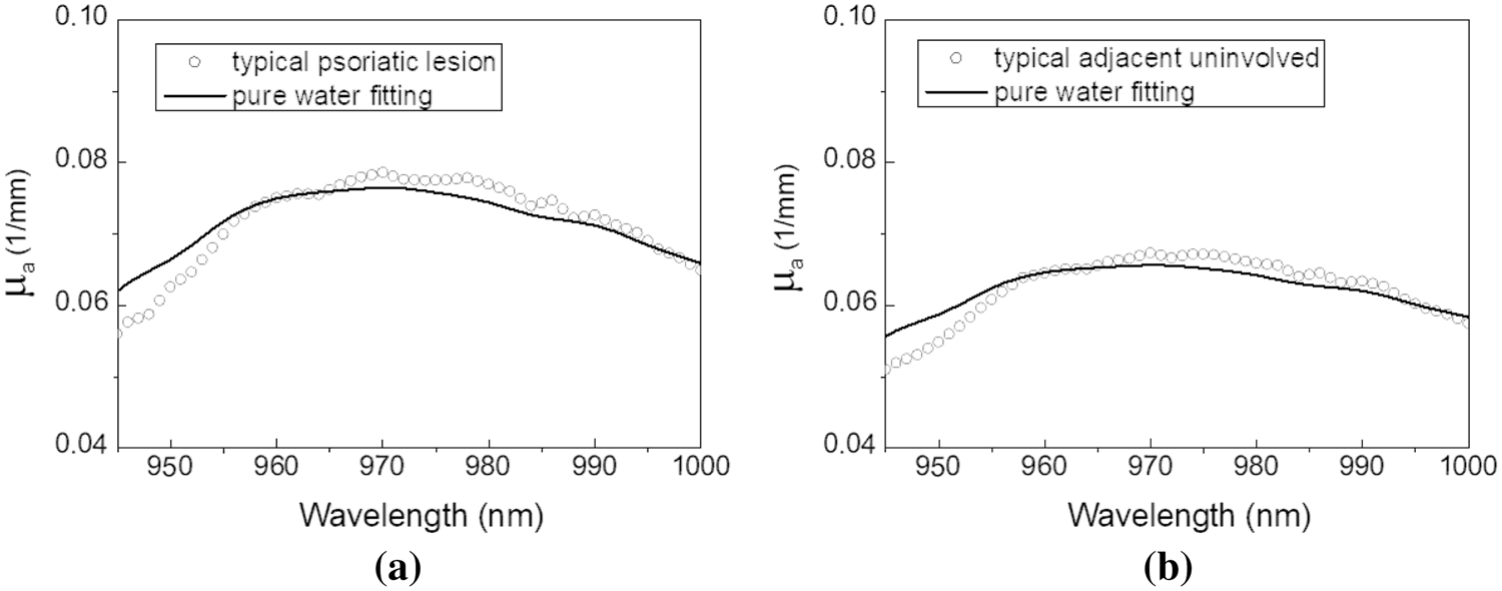
Typical skin absorption spectra of psoriatic lesion site and its adjacent uninvolved site of the representative psoriasis subject at the “second overtone” of water absorption. The absorption spectra of (**a**) the psoriatic lesion site and (**b**) its adjacent uninvolved site are shown in dotted lines. Best pure water absorption fitting to the skin absorption spectra are shown in solid lines.

To understand the characteristics of psoriasis absorption spectra, a representative subject with typical psoriatic lesions was selected from the 21 subjects recruited for the analyses in this subsection. The typical absorption spectra of the psoriatic lesion site and its adjacent uninvolved skin site of a subject near 970 nm are displayed in Fig. 2a,b, respectively. The absorption and reduced scattering spectra of the psoriatic lesions of all 21 psoriasis subjects at this second overtone water absorption band are depicted in the Supplementary Fig. S1 online. It can be seen in Fig. 2 that the magnitude of absorption of psoriatic lesion is slightly larger than that of adjacent uninvolved skin. This phenomenon was universal for all psoriasis subjects recruited in this study and we believe this was caused by the higher skin blood content of psoriatic lesion than the uninvolved skin. The best pure water spectrum fitting to the absorption spectra are displayed as solid lines in the figures. It can be observed in Fig. 2 that the absorption spectra of the lesion site as well as the adjacent uninvolved site deviate from the pure water absorption spectrum. This phenomenon is similar to the bound water phantom measurement results displayed in Fig. 1a. The pure water fitting residuals of the lesion and uninvolved skin absorption spectra between 940 and 1000 nm were 0.55 and 0.47, respectively. In this case, the fitting residual of lesion site is higher than that of uninvolved skin.

**Figure 3:**
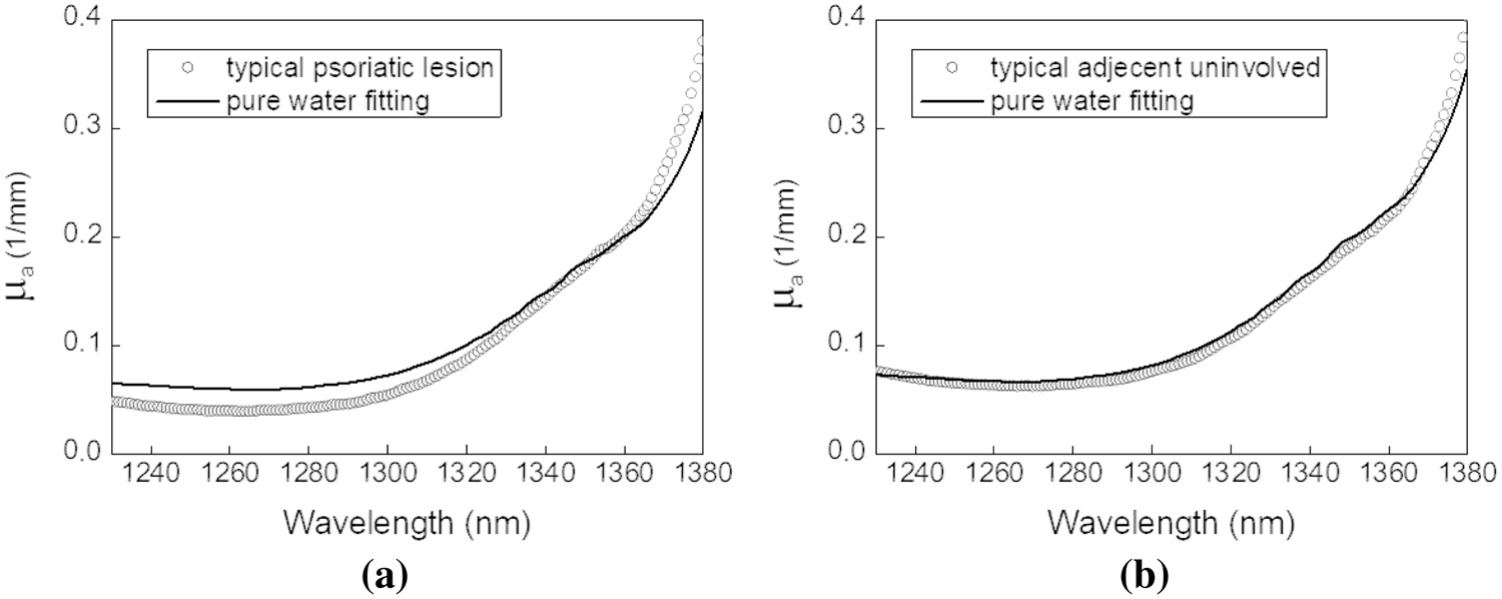
Typical skin absorption spectra of psoriatic lesion site and its adjacent uninvolved site of the representative psoriasis subject at the “first overtone” of water absorption. The absorption spectra of (**a**) the psoriatic lesion site and (**b**) its adjacent uninvolved site are shown in dotted lines. Best pure water absorption fitting to the skin absorption spectra are shown in solid lines.

The typical psoriatic lesion and adjacent uninvolved skin absorption spectra near 1300 nm and their best pure water fitting spectra are depicted in Fig. 3. The absorption and reduced scattering spectra of the psoriatic lesions of all 21 psoriasis subjects at this first overtone water absorption band are depicted in the Supplementary Fig. S2 online. In general, at this first water absorption overtone band, the absorption spectra of both skin sites demonstrate water absorption peak broadening, resembling the bound water phantom measurement results shown in Fig. 1b. The pure water fitting residuals of the psoriasis and normal skin absorption spectra between 1230 and 1380 nm were 7.61 and 2.77, respectively. It is apparent that the fitting residual values at the first overtone band are much larger than those at the second overtone band. Besides, qualitative observation of the spectra difference between psoriasis and normal skin (Fig. 2a,b vs. Fig. 3a,b) reveals that the difference is more prominent at the first overtone band. In addition, we noted that the skin absorption spectra of normal subjects (not depicted here) were comparable to those of uninvolved skin as displayed in Figs. 2b and 3b.

#### Statistical analyses of skin absorption spectra

**Figure 4:**
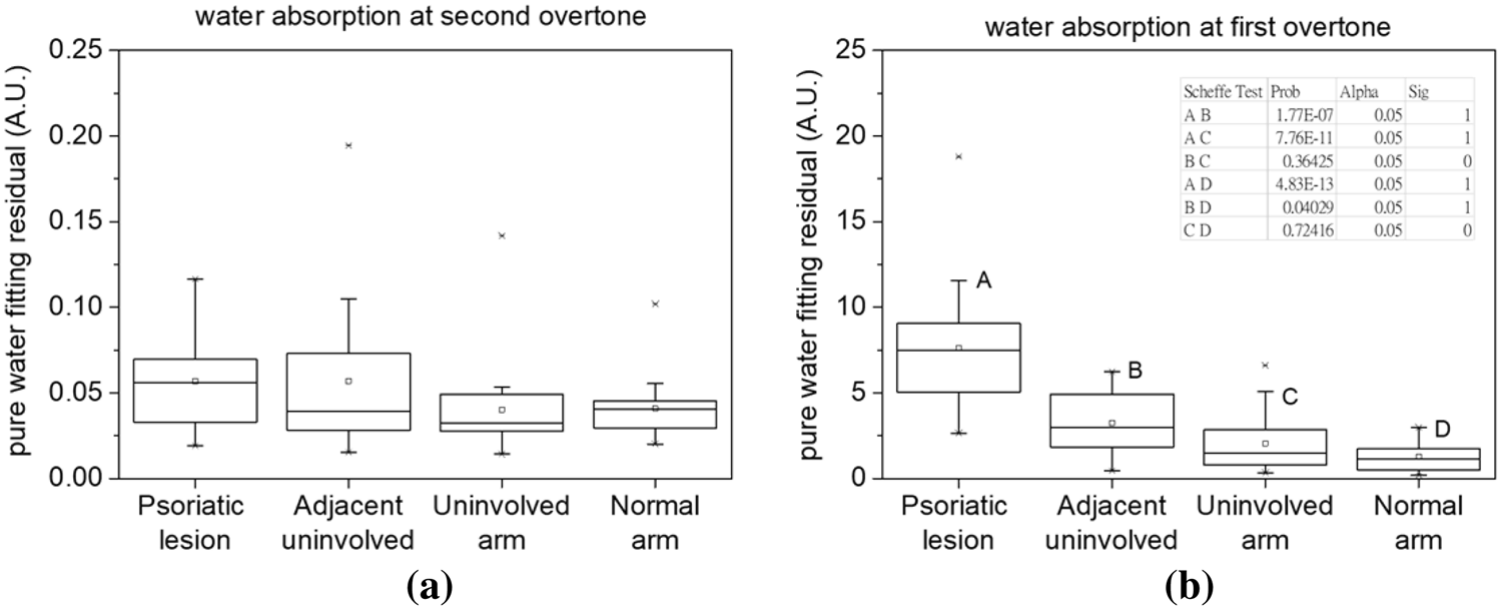
Statistics summary of pure water fitting residuals of the four measurement sites at the second and first overtone bands of water absorption. (**a**) Box-and-whisker plots shows that the means of pure water fitting residuals near 970 nm of the four sites are comparable. The *P*-value of one-way ANOVA was 0.21, indicating that the population means were not significantly different. (**b**) Box-and-whisker plots shows that the means of pure water fitting residuals near 1300 nm of the four sites. The *P*-value of one-way ANOVA was 9.65E−15, indicating that the population means were significantly different. Inset table shows Scheffѐ test *P*-values between any two groups, and Sig equals to 1 indicates that the difference of the means is significant at the 0.05 level.

We carried out one-way ANOVA to investigate the differences between the pure water fitting residuals of psoriatic lesion sites, the adjacent uninvolved sites and uninvolved upper inner arms of the 21 psoriasis subjects, and normal upper inner arms of the 21 normal subjects. Box-and-whisker plots displayed in Fig. 4 summarized the statistics results of one-way ANOVA. We first noted that, in Fig. 4a, the distribution of fitting residuals of lesion sites, adjacent uninvolved sites, uninvolved upper inner arms are comparable. The *P*-value of one-way ANOVA of the four groups was 0.21, indicating that the population means were not significantly different. It can be deduced that although at second overtone band the bound water effect can be observed in the spectrum analysis such as those shown in Fig. 2, the fitting residuals at this band could not be used as an effective parameter for distinguishing the lesion site from the uninvolved skin of a psoriasis subject nor from the normal skin. On the other hand, it can be seen in Fig. 4b that the distribution of pure water fitting residuals of lesion sites is significantly higher than those of the other three sites. The *P*-value of one-way ANOVA of the four groups was 9.65E−15, indicating that the population means were significantly different. By carrying out Scheffѐ tests, we found that fitting residuals of psoriatic lesion at the first overtone band was significantly higher than the other three sites, and the uninvolved skin adjacent to the lesion site had significantly higher fitting residuals than those of upper inner arm skin of normal subjects. At this first overtone band, the fitting residuals of the upper inner arm skin of psoriasis and normal subjects were not significantly different. Our results suggest that pure water fitting residual near the first water absorption overtone could be an effective indicator of psoriasis.

In addition, we found that Pearson correlation coefficients between the PASI parameters listed in Supplementary Table S1 online, including erythema, thickness, and scaling, and the fitting residual of water absorption at first overtone band were 0.22, 0.31, and 0.46, respectively. The degree of scaling reflects the status of skin barrier and this parameter had the highest correlation coefficient among the three PASI parameters. This result supports our speculation that the pure water fitting residual at the first overtone could be an effective indicator of the water bonding status in the skin.

#### Reduced scattering

**Figure 5:**
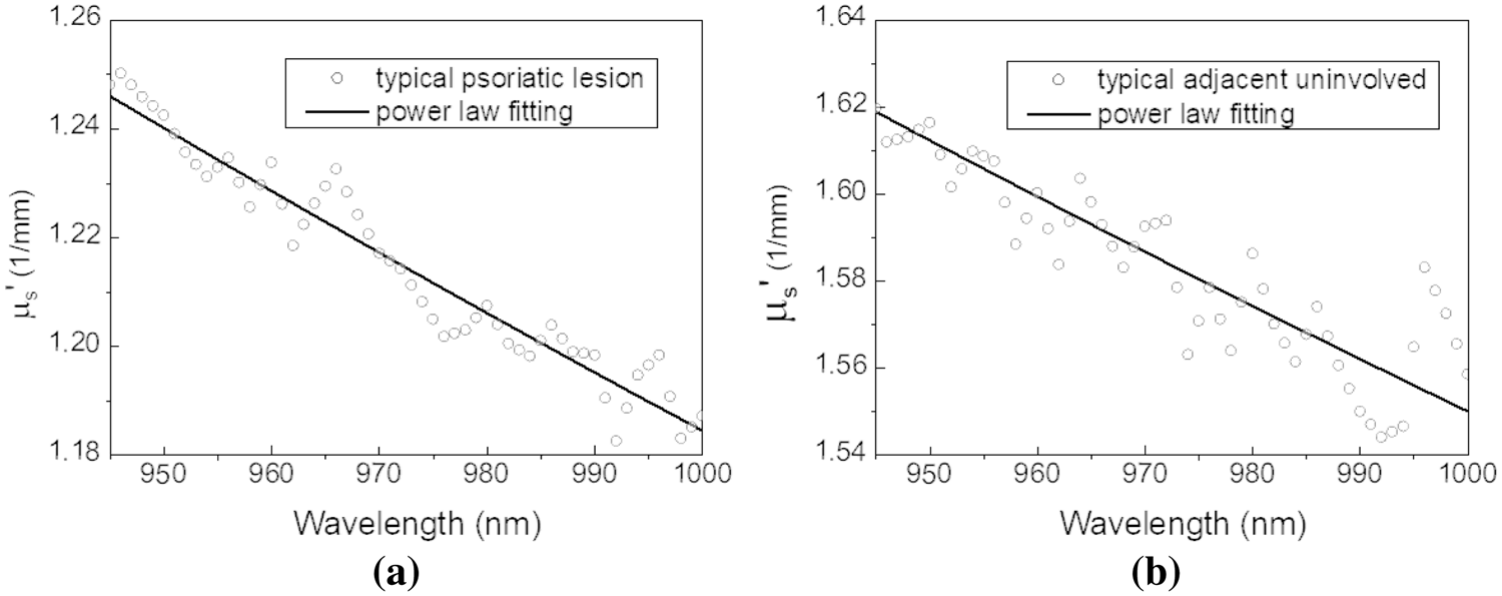
Typical skin reduced scattering spectra of psoriatic lesion site and its adjacent uninvolved site of the representative psoriasis subject at the “second overtone” of water absorption. The reduced scattering spectra of (**a**) the psoriatic lesion site and (**b**) its adjacent uninvolved site are shown in dotted lines. Best power law fitting to the skin reduced scattering spectra are shown in solid lines.

The reduced scattering coefficients of the typical psoriatic lesion and its adjacent uninvolved site of the representative subject at the second overtone of water absorption are illustrated in Fig. 5a,b, respectively. The reduced scattering coefficients of uninvolved site are generally higher than the lesion counterpart. This observation agrees with what we have reported earlier where the analyzed wavelength range was from 500 to 900 nm^15^. The scattering power law (μ_s_' = a*λ^−b^) was employed to smooth the reduced scattering spectra in the 940–1000 nm range, and the fitting results are shown as solid lines in Fig. 5. The magnitude “a” and wavelength exponent “b” of the scattering power law fitting were 315.565 and 0.808, respectively, for the typical lesion shown in Fig. 5a, and were 330.316 and 0.777, respectively, for the adjacent uninvolved site shown in Fig. 5b.

**Figure 6:**
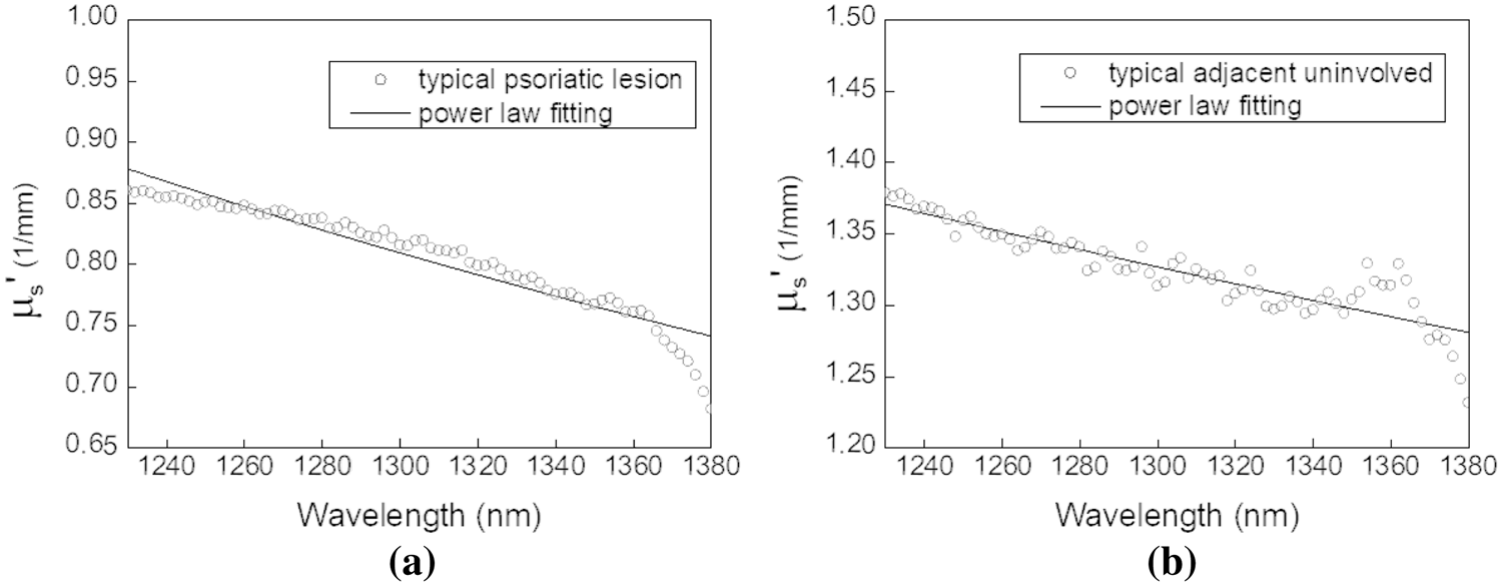
Typical skin reduced scattering spectra of psoriatic lesion site and its adjacent uninvolved site of the representative psoriasis subject at the “first overtone” of water absorption. The reduced scattering spectra of (**a**) the psoriatic lesion site and (**b**) its adjacent uninvolved site are shown in dotted lines. Best power law fitting to the skin reduced scattering spectra are shown in solid lines.

Consistent with the results observed in the second overtone band, the reduced scattering coefficients of psoriatic lesion were lower than those of uninvolved site at the first overtone band, as can be seen in Fig. 6. Due to strong water absorption near the first overtone band, the scattering coefficients at 1370 nm drop drastically. The magnitude “a” and wavelength exponent “b” of the scattering power law fitting in the 1230–1380 nm range were 257,912.881 and 1.762, respectively, for the typical lesion site shown in Fig. 6a, and were 484.744 and 0.823, respectively, for the adjacent uninvolved site shown in Fig. 6b.

#### Statistical analyses of skin reduced scattering spectra

**Figure 7:**
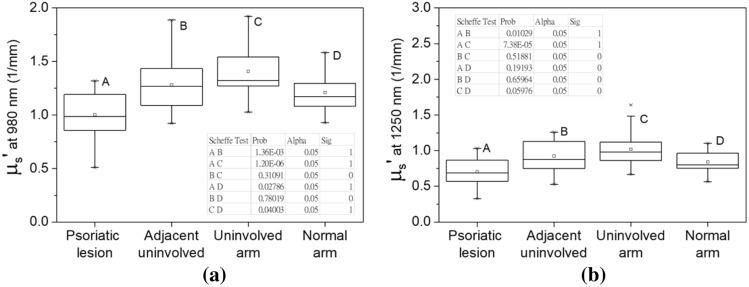
Box-and-whisker plots of reduced scattering coefficients of the four measurement sites at 970 nm and 1300 nm. (**a**) At 970 nm, the *P*-value of one-way ANOVA was 6.61E−7, indicating that the reduced scattering coefficients were significantly different. Inset table shows Scheffѐ test *P*-values between any two groups, and Sig equals to 1 indicates that the means of groups are significantly different at the 0.05 level. (**b**) Box-and-whisker plots of 1300 nm reduced scattering coefficients of the four sites. The *P*-value of one-way ANOVA was 3.76E−5, indicating that the population means were significantly different. Inset table shows Scheffѐ test *P*-values between any two groups.

One-way ANOVA was performed to investigate the reduced scattering coefficients differences between various skin sites of the 21 psoriasis subjects and 21 normal subjects. We only selected the reduced scattering coefficients at 970 nm and 1300 nm for the analyses. The analysis results are summarized in the box-and-whisker plots illustrated in Fig. 7. One-way ANOVA indicated that the 970 nm reduced scattering coefficients of the four measurement sites were significantly different (*P* = 6.61E−7). Employing further Scheffѐ tests revealed that the psoriatic lesions had significantly lower 970 nm reduced scattering coefficients than those of adjacent uninvolved skin, uninvolved upper inner arm skin, and normal upper inner arm skin. The complete Scheffѐ test results are listed in the inset of Fig. 7a. On the other hand, at 1300 nm wavelength, the reduced scattering coefficients of the uninvolved arm group had highest mean as shown in Fig. 7b. This data distribution is qualitatively similar to that of 970 nm reduced scattering coefficients shown in Fig. 7a. One-way ANOVA indicated that the 1300 nm reduced scattering coefficients of the four measurement sites were significantly different (*P* = 3.76E−5). Scheffѐ test results indicate that at 1300 nm the reduced scattering coefficients of psoriatic lesions were not significantly lower than those of normal skin (*P* = 0.19), which is different from the results shown in Fig. 7a. At 1300 nm wavelength, the reduced scattering coefficients of psoriatic lesion were significantly lower than those of adjacent uninvolved skin and uninvolved upper inner arm skin (*P* = 0.01 and *P* = 7.38E−5). Our results suggest that while the light scattering property of skin at either the first or the second overtone of water absorption band could be used for psoriasis diagnosis, using second overtone band would be more effective than the first overtone band. This is reasonable since the skin reduced scattering coefficient decreases as wavelength increases, and the difference between the light scattering property of psoriatic lesion and normal skin would be more prominent at the shorter wavelengths.

We further analyzed the values of the magnitude “a” and the exponent “b” of the scattering power law that fit to the skin reduced scattering spectra. Surprisingly, we found one-way ANOVA results that neither “a” nor “b” values derived from 940 to 1000 nm or 1230 to 1380 nm showed significant differences between the measurement sites. We observed that the span of “a” and “b” values of the 21 psoriatic lesion sites were quite large as compared with those of the other sites. The “a” and “b” values in the scattering power law were linked to the scatterers’ number density and average size, respectively^25^. In addition, it has been indicated that the light scattering of biological tissues is majorly induced by the organelles and membranes of cells which provide index of refraction perturbation^25^. Our finding suggests that the size of fine light-scattering structures in the epidermis of psoriasis skin have great variation as compared with those of the adjacent uninvolved or normal skin.

## Discussion

Healthy skin stratum corneum is composed of corneocytes and intercellular lipids, such as free fatty acids, cholesterol, and ceramides, and their arrangement has been described as the “brick and mortar” model. In addition to the function of reducing the water out-flux from viable cells in epidermis and dermis, stratum corneum itself contains around 30% of water which mainly present within the corneocytes^26^. The corneocytes are filled with crosslinked keratin and many water domains are presented between keratin filaments^26^. In a recent study employing in-vivo confocal Raman spectroscopy, it was reported that at the intermediate layers (30–70% depth) of stratum corneum, water molecules are bound to keratin filaments^27^. Beneath the stratum corneum layer, the water content of the highly hydrated viable epidermis could reach above 70%^28^. Since the protein structure in viable epidermis is loose, the majority of water molecules in this region are not bound. Water molecules that are not bound to macromolecules are able to diffuse from the skin to the environment, such phenomenon would be more prominent when the epidermal barrier function is impaired.

Clinical evidence suggests that epidermal barrier function defects play a role in psoriasis pathogenesis^29^. In psoriasis, the epidermal keratinocytes divide much faster than normal and could have a disorganized stratum corneum layer which would result in compromised skin barrier function and increased water out-flux. Grice et. al. found that the barrier function of the psoriatic lesion is defective and transepidermal water loss (TEWL) is increased compared with normal healthy skin^30^. The compromised skin barrier function has been recognized as the consequence of disturbed and altered distribution of stratum corneum ceramides^31^.

The probing region of our DRS configuration contains epidermis and upper dermis of human skin, and thus our system is sensitive to the chromophore and structural variation in this skin region^32^. We found that, in the wavelength range from 500 to 900 nm, while the oxygenated and deoxygenated hemoglobin concentrations of psoriatic skin were distinct from those of normal sites, other skin functional parameters, such as melanin, collagen, and reduced scattering coefficients, did not show statistically significant difference between the psoriatic and normal sites^15^. However, using the skin hemoglobin concentration derived from DRS alone is difficult in achieving effective psoriasis diagnosis or monitoring.

Bound water fraction in tissue has been used as an evaluation parameter for disease state, such as breast cancer^24^, and has also been used for investigation of epidermal water–lipid interaction and water holding capability^33,34^. In this study, we discovered that the skin water absorption spectrum contains prominent features that can be used for effective differentiation between normal skin and psoriatic skin. Our results shown in Fig. 4b suggests that the bound water fraction at psoriatic lesion site was significantly higher than those of the other three sites. It can be inferred that due to impaired skin barrier function of psoriatic skin, free water molecules are diffused into environment and thus the measured water absorption spectrum comes from the contribution of bound water left in the stratum corneum and epidermis. This phenomenon can be supported by the results reported by Bouwstra et al., where they found that for normal skin in dry condition (hydration level of 18–26% wt/wt), only bound water is present in the stratum corneum^26^.

On the other hand, since the capillaries were dilated in the upper dermis of the psoriatic skin, the blood volume, and thus the free water volume, in the dermis of psoriatic skin would be higher than that of normal skin. Our previous experimental and numerical study results indicate that while the probing region of our DRS system covers epidermis and dermis, the variation in epidermis would produce much more impact on the detected signal than that in dermis^35^. Thus, we speculate that although the bound water and free water are both elevated in the psoriatic skin, the light absorption features of bound water in epidermis dominate the diffuse reflectance spectrum captured by our DRS system as observed in Fig. 3a.

Light scattering property of biological tissues has been used for monitoring tissue morphology variation^36–38^. Our findings demonstrated in Fig. 7 indicate that the psoriatic lesion skin is less capable in scattering light than the uninvolved and normal skin in general, and this fact could result in the dull appearance of psoriatic lesion. This observation agrees with work of Welzel et al., where they used optical coherence tomography to investigate psoriasis skin and found that the light scattering of involved skin was lower than those of uninvolved skin^39^. Besides, various studies, through the use of microscopy, Fourier-transform infrared spectroscopy, and Raman scattering spectroscopy, have pointed out that psoriatic skin has a reduction in the definition of skin layer boundaries and the cell arrangement is less organized as compared to normal skin^16,40^. These reports support our findings which suggest that the skin structure of psoriatic lesion is distinct from uninvolved or normal skin.

Both tissue scatters and absorbers would affect the shape and intensity of diffuse reflectance spectra. DRS is a model driven optical technique that can effectively extract tissue absorption and scattering spectra from a measured diffuse reflectance spectrum. Thus, the influence of tissue scatters in the absorption spectrum used for bound water analysis would be minimized in our DRS system. Our current physics model used for diffuse reflectance spectra analyses assumes that tissue is a homogeneous medium and does not account for the layer structure of skin. In addition, from Monte Carlo simulations, it was found that the interrogation depth of our DRS system was in the range from 400 to 600 µm in this study. Therefore, had the stratum corneum thickness of skin varied, the recovered skin absorption and scattering spectra would certainly be affected.

It should be noted that the factors that could affect the diffuse reflectance spectra in the psoriatic skin, in addition to epidermal thickness mentioned above, could also include the presence of inflammatory cells in the epidermis and dermis, the modification of capillary loop and hemoglobin concentration in the upper dermis. While these factors could affect the skin scattering spectrum, they do not possess known absorption characteristics at the first overtone band of water absorption. This warrants further numerical and clinical studies to investigate the effect of these factors on the skin absorption spectrum at the first overtone band.

In conclusion, in this study, we found that near the first overtone of water absorption, the variations in skin bound water and cell organization could be monitored using a simple diffuse reflectance spectroscopy system. In this pilot study, it can be seen that the skin absorption spectrum fitting residuals and light scattering properties in the 1230–1380 nm range are effective parameters for differentiating psoriatic lesion from uninvolved and normal sites. We will carry out further studies to understand the linking between these parameters and the skin hydration status, and whether they could be utilized to reflect the psoriasis treatment efficacy or to monitor the water loss of normal skin. In addition, we will plan the integration of proper light emitting light diodes in the 1230–1380 nm band and photo diodes into a handheld DRS system based on our previous experience in building a handheld DRS system^15^, and test its feasibility of objectively evaluating the epidermal barrier function in the psoriasis skin.

## Materials and methods

### Measurement setup

**Figure 8:**
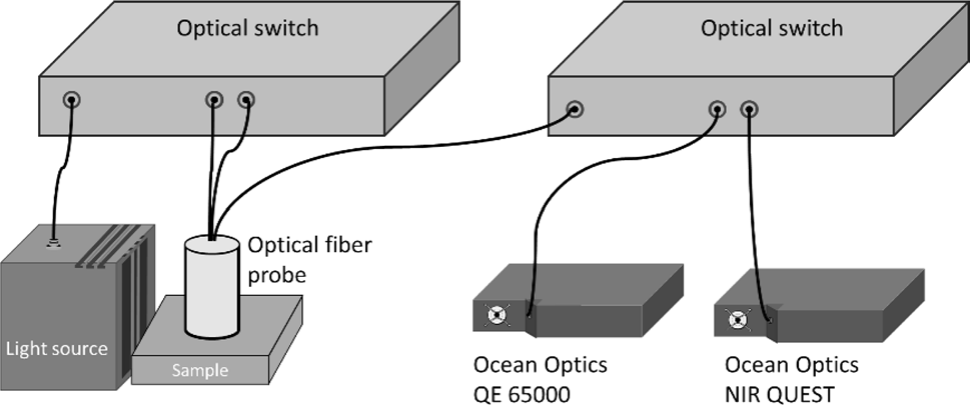
Schematic diagram of the DRS system setup employed in this study. Two optical switches were used in the system where one was used for switching light source to one of the source fibers to deliver light to the sample, and the other was used to switch the diffuse reflectance collected by the detector fiber to one of the spectrometers of different wavelength detection ranges.

Our DRS system consists of a broadband light source (EQ-99X, Energetiq, USA), two 1 × 2 optical fiber switches (Piezosystem Jena, Germany) and two spectrometers (QE65000 and NIR QUEST, Ocean Optics, USA). The broadband light source emits light in the range from 170 to 2400 nm and its optical power varies with wavelength and is in the range from 1 to 30 mW/(mm^2^ sr nm). The system is capable of collecting skin diffuse reflectance in the wavelength range from 500 to 1600 nm, and its setup is shown in Fig. 8. A custom optical fiber probe (Leoni, Germany) containing three aligned fibers was employed for light delivery and collection. The diameter and numerical aperture of the three fibers were 400 μm and 0.22 respectively, and the distance between any two adjacent fibers was 1 mm. A custom MATLAB (MathWorks, USA) routine was developed to control the optical fiber switches and the spectrometers to collect diffuse reflectance at the two source-to-detector separations (SDSs).

### Phantoms for observing bound water effect

The O–H bond of pure water absorbs light strongly at several wavelengths. Among the absorption peaks of O–H bond, the 970 nm (second overtone) and 1470 nm (first overtone) peaks are induced by the combination of symmetric stretch and asymmetric stretch of O–H bond and are prominent in short wave near infrared range. As mentioned earlier, the absorption spectrum of water molecule would be influenced by the nearby macromolecules. To study this bound water effect, Chung et al. fabricated phantoms by mixing water, gelatin powder, and light scattering agent, and they found that the water absorption spectrum at the second overtone varied due to the presence of gelatin powder^24^. To understand the bound water effect near the first and second overtone of water absorption, we followed the bound water phantom recipe provided by Chung et al. and dissolved 8 g of gelatin powder and 30 ml 20% Lipofundin (B. Braun Melsungen AG, Germany) in 300 ml of pure water. An integrating sphere system was employed to measure the transmittance and reflectance of the bound water phantom to determine its optical properties using the inverse adding doubling algorithm developed by Prahl^41^.

### In vivo human skin measurements

In this study, we recruited 21 subjects diagnosed with psoriasis by dermatologists (average age: 47.1 years; standard deviation: 14.8 years) and 21 normal subjects (average age: 39.5 years; standard deviation: 12.9 years) at National Cheng-Kung University Hospital, Taiwan. The protocol was approved by the Institutional Review Board of National Cheng Kung University Hospital, Taiwan (No. ER-100-332). Written informed consents were obtained from all the subjects in the study. The study was conducted in accordance with the latest revision of the Declaration of Helsinki. For each subject with psoriasis, we measured a psoriatic lesion site selected by dermatologists, and an adjacent uninvolved site (2–3 cm apart from the edge of lesion). The three parameters of PASI score, erythema, thickness and scaling, were rated based on the lesion pictures and the average of the scores from four dermatologists are listed in Supplementary Table S1 online. Since the selected psoriatic lesion sites are not necessarily located at the same region for all subjects, to reasonably compare the skin condition of all subjects, an uninvolved site at the middle of right upper inner arm, a typical site of lesser sun exposure, of each subject was also measured. For normal subjects, measurements were carried out only at the middle of right upper inner arm. Five measurements were carried out at each site, and the optical fiber probe was removed from the site after each measurement.

### Skin optical properties recovery

We used an artificial neuron network (ANN) model to recover the tissue optical properties from the collected skin reflectance. The model construction and validation has been described in detail elsewhere^42^. In short, the diffuse reflectance collected using the probing geometry mentioned in 2.3 at various tissue optical properties were calculated using the Monte Carlo method. In the Monte Carlo simulations, the tissue sample was assumed to be homogeneous with index of refraction of 1.45, and the sample optical properties varied from 0.001 to 1.00 mm^−1^ in a 0.001 mm^−1^ step for absorption *µ*_a_ and varied from 0.10 to 3.00 mm^−1^ in a 0.10 mm^−1^ step for reduced scattering *µ*_s_'. The Henyey-Greenstein phase function with anisotropy factor of 0.8 was employed in all simulations. An ANN model was trained with the calculated diffuse reflectance database using the MATLAB training function “trainbr”, and the model can determine tissue absorption and reduced scattering spectra from the measured diffuse reflectance spectra collected at 1 and 2 mm SDSs. In this study, the ANN model was used to recover the skin absorption and reduced scattering spectra in the wavelength range from 940 to 1000 nm, as well as from 1230 to 1380 nm to investigate the water state of psoriatic and normal skin.

### Absorption spectrum fitting residual for bound water evaluation

Based on the fact that the bound water absorption spectrum is different from that of pure water, the recovered absorption spectra of skin or phantoms were compared to the pure water absorption spectrum. To quantitatively perform the comparison, we used curve fitting method to determine the best pure water absorption spectrum fit to the sample absorption spectra. The pure water absorption spectrum at 25 ℃ was obtained from a transmission spectrophotometer (U-4100, Hitachi, Japan). We employed the “lsqcurvefit” function in MATLAB to perform the curve fitting. The fitting residual returned from the function was divided by the number of spectral data points. These values were then multiplied by 100 and displayed in Figs. 3, 4, and 5.

### Statistical analysis

To compare the absorption spectra fitting residuals of the four measurement sites, namely, psoriatic lesion, adjacent uninvolved and uninvolved upper inner arm of the 21 psoriasis subjects, and normal upper inner arm of the 21 normal subjects, one-way analysis of variance (ANOVA) was performed using OriginPro2017 software (OriginLab, USA). Box-and-whisker plots were generated to describe the statistics summary. In the plots, the length of the box represents the distance between the 25th and 75th percentiles, the symbol in the box represents the group mean, the horizontal line in the box represents the group median, the whiskers issuing from the box extend to the group minimum and maximum values, and the outliers are shown in asterisks. The statistical significance was defined at *P* < 0.05. If the one-way ANOVA rejected the null hypothesis of equal means of all groups, Scheffѐ tests would be carried out to investigate the difference of the means of any two groups.

## Supplementary Information

Supplementary Information
