# Supplementary material for: Investigation of water bonding status of normal and psoriatic skin in vivo using diffuse reflectance spectroscopy
Source: Sci Rep. 2021 Apr 26;11:8901. doi: 10.1038/s41598-021-88530-y (PMC8076238; doi:10.1038/s41598-021-88530-y)
Supplement: Supplementary file 1 — Supplementary Information [file 41598_2021_88530_MOESM1_ESM.docx]

Supplementary figures


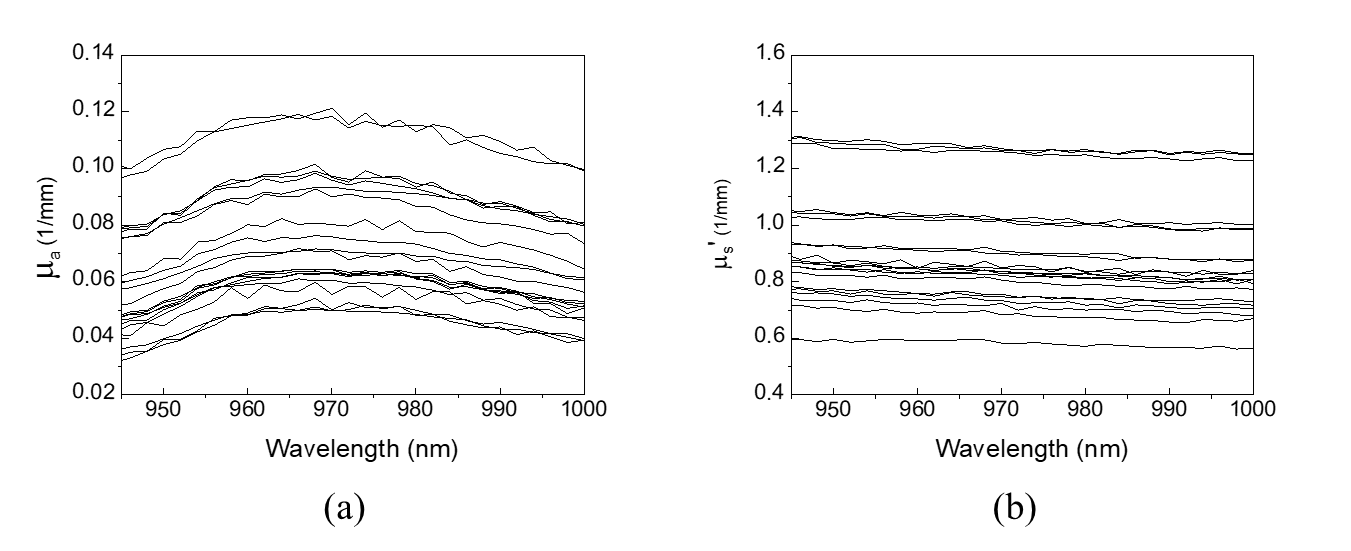


Fig. S1. (a) Absorption spectra, and (b) reduced scattering spectra of psoriatic lesions of 21 psoriasis subjects in the 940-1000 nm wavelength region.


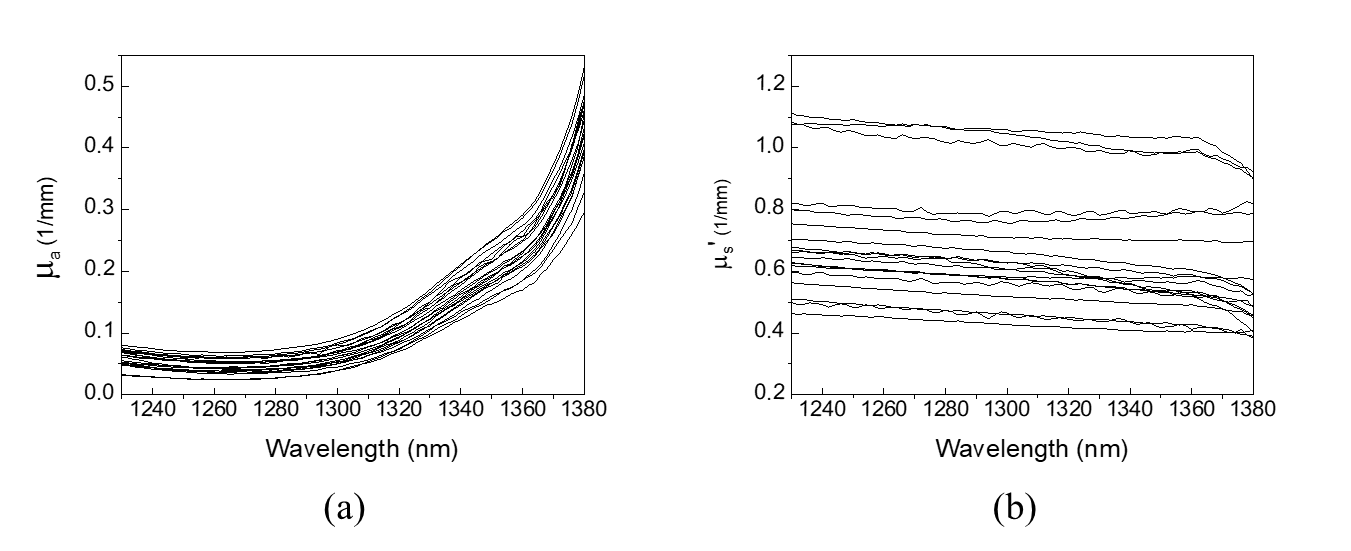


Fig. S2. (a) Absorption spectra, and (b) reduced scattering spectra of psoriatic lesions of 21 psoriasis subjects in the 1230-1380 nm wavelength region.

Supplementary table

Table S1. The average scores of the erythema, thickness, and desquamation of the psoriatic lesion sites of 21 psoriasis patients rated by four physicians.

| Subject  (site) | Scores | | | Subject  (site) | Scores | | |
| --- | --- | --- | --- | --- | --- | --- | --- |
|  | Erythema | Thickness | Desquamation |  | Erythema | Thickness | Desquamation |
| P01  (leg) | 2.25 | 1.00 | 0.50 | P12  (leg) | 1.50 | 1.25 | 0.75 |
| P02  (leg) | 2.00 | 0.50 | 0.00 | P13  (leg) | 2.25 | 0.75 | 1.00 |
| P03  (leg) | 1.25 | 1.25 | 1.25 | P14  (leg) | 2.00 | 0.75 | 1.00 |
| P04  (arm) | 3.00 | 1.75 | 2.00 | P15  (trunk) | 1.75 | 0.75 | 1.25 |
| P05  (leg) | 1.75 | 2.50 | 1.25 | P16  (head) | 1.75 | 1.00 | 0.75 |
| P06  (arm) | 1.25 | 2.00 | 1.75 | P17  (arm) | 2.50 | 1.25 | 1.75 |
| P07  (leg) | 2.00 | 1.25 | 1.75 | P18  (leg) | 3.25 | 2.00 | 3.00 |
| P08  (arm) | 1.75 | 2.50 | 2.75 | P19  (arm) | 0.75 | 2.25 | 2.50 |
| P09  (leg) | 0.50 | 0.50 | 0.75 | P20  (leg) | 2.25 | 1.00 | 1.50 |
| P10  (trunk) | 2.75 | 2.50 | 1.00 | P21  (trunk) | 1.25 | 0.00 | 0.25 |
| P11  (leg) | 2.50 | 1.75 | 1.50 |  |  |  |  |
